# Digit Ratio (2D:4D): A Biomarker for Prenatal Sex Steroids and Adult Sex Steroids in Challenge Situations

**DOI:** 10.3389/fendo.2014.00009

**Published:** 2014-01-30

**Authors:** John Manning, Liam Kilduff, Christian Cook, Blair Crewther, Bernhard Fink

**Affiliations:** ^1^Department of Psychology, Northumbria University, Newcastle upon Tyne, UK; ^2^Applied Sports Technology, Exercise and Medicine Research Centre, Swansea University, Swansea, UK; ^3^School of Sport, Health and Exercise Science, Bangor University, Gwynedd, UK; ^4^Hamlyn Centre, Imperial College, London, UK; ^5^Department of Biological Personality Psychology and Assessment, University of Göttingen, Göttingen, Germany

**Keywords:** digit ratio, testosterone, estrogen, performance, aggression, organizing effects, activating effects

## Abstract

Digit ratio (2D:4D) denotes the relative length of the second and fourth digits. This ratio is considered to be a biomarker of the balance between fetal testosterone (T) and estrogen (E) in a narrow window of early ontogeny. Evidence for this assertion is derived from direct and indirect measures of prenatal hormonal exposure (in experimental animals, via amniotic fluid samples and in the study of sex-typical traits) in relation to 2D:4D. In contrast, the relationships between 2D:4D and levels of sex steroids in adults are less clear, as many correlational studies of 2D:4D and adult sex steroids have concluded that this association is statistically non-significant. Here, we suggest that in order to understand the link between 2D:4D and sex hormones, one must consider both fetal organizing and adult activating effects of T and E. In particular, we hypothesize that 2D:4D correlates with organizing effects on the endocrine system that moderate activating effects in adulthood. We argue that this is particularly evident in “challenging” conditions such as aggressive and sexual encounters, in which individuals show increased levels of T. We discuss this refinement of the 2D:4D paradigm in relation to the links between 2D:4D and sports performance, and aggression.

## Introduction

Digit ratio (or 2D:4D) is the relative lengths of the second digit (the “index” finger) and the fourth digit (the “ring” finger). It has been known for many years that 2D:4D varies according to sex, such that males tend to have longer fourth digits relative to second digits (low 2D:4D) than females [high 2D:4D; see Ref. (1)]. The effect size of this sex difference is small to moderate (Cohen’s *d* is about 0.50), and 2D:4D attracted little attention until 1998, when it was suggested that a balance of fetal testosterone (FT) and fetal estrogen (FE) influences the formation of 2D:4D, such that low 2D:4D indicates high FT and low FE and high 2D:4D indicates low FT and high FE [(2); see Ref. (3) for a detailed development of the hypothesis]. Following this suggestion, there was a marked increase in the number of 2D:4D studies, from 1 in 1998 to 51 in 2007, and from 2008 to 2010 the numbers of studies have averaged about 60 papers per year (4).

The initial statement of the hypothesis that 2D:4D is a morphological correlate of FT and FE was derived from two data sets (2, 3) reporting the following relationships: (i) age effects; a longitudinal sample of 800 children and adults aged from 2 to 25 years showed a sex difference in 2D:4D, such that males tended to have lower 2D:4D than females, and mean 2D:4D did not change significantly with age. Therefore, it is likely that the sexual dimorphism is determined early in ontogeny, probably *in utero*, (ii) hormone and fertility effects; data from 131 participants (69 males) attending an infertility clinic showed that high 2D:4D was linked to germ cell failure, low sperm numbers, and high levels of estrogen (E), while low 2D:4D was linked to high testosterone (T) and high sperm numbers. It was concluded that the sex difference in 2D:4D was likely to be determined *in utero* by a balance of T and E and that adult levels of these hormones echo prenatal concentrations of sex steroids. Subsequent studies have provided support for the link between 2D:4D and FT and FE, but have not confirmed the associations between 2D:4D and adult concentrations of T and E.

## 2D:4D and Prenatal Testosterone and Estrogen

The sex difference in 2D:4D is now well understood [see Ref. (5) for a meta-analysis]. It is found in fetuses as early as the end of the first trimester (6, 7) and although it may change postnatally as the fingers grow, this change is in the form of a gentle increase in 2D:4D (8, 9). Fetal levels of T are sexually dimorphic with male fetuses having higher T than female fetuses. Therefore, this pattern of prenatal determination of the sexual dimorphism in 2D:4D is consistent with – but does not prove that – the sex difference in 2D:4D reflects fetal levels of sex steroids.

It is difficult to measure the effect of FT and FE on 2D:4D in humans directly because of ethical constraints. This is why a readily measured indirect correlate of the balance between FT and FE (such as 2D:4D) is valuable in investigating organizing effects on sex-typical traits. However, the difficulties associated with measuring fetal hormones and fetal 2D:4D means that the link between FT/FE and 2D:4D is not easily demonstrated. Attempts to investigate this relationship may be considered as of two kinds, i.e., correlational studies and experimental studies.

Correlational studies have considered relationships between 2D:4D and sexually dimorphic physical and behavioral traits that are thought to be linked to FT and FE. There are many of these; here we focus on some of the more important ones, i.e., those that are very likely to be affected by FT and/or FE. Congenital adrenal hyperplasia (CAH) is a trait associated with an increase in the size of the fetal adrenal glands and an elevated level of fetal androgens. To date, there have been four studies of CAH and 2D:4D. All have shown a tendency for low 2D:4D (i.e., “masculinized” 2D:4D) to be linked to CAH and in three such studies, the effects were significant [see Ref. (5) for a meta-analysis of these studies]. In contrast to CAH patients, individuals with Klinefelter’s syndrome (males with 47 chromosomes, including XXY) have low fetal androgen levels. In Klinefelter patients, 2D:4D is significantly higher (i.e., “feminized” 2D:4D) than that of the population norm (10). This pattern of “feminized” 2D:4D has also been found in individuals who suffer from androgen insensitivity (11), i.e., a clinical condition that results in a partial or complete inability of cells in their response to androgens. All these studies have focused on FT. However, Lutchmaya et al. (12) obtained both FT and FE concentrations from amniotic fluid samples in order to investigate relationships with 2D:4D. It was found that 2D:4D of neonates was related to a balance of FT and FE, such that high FT and low FE were linked to “masculinized” 2D:4D.

The link of digit ratio to CAH, Klinefelter’s syndrome, and androgen insensitivity is strong evidence for a link between 2D:4D and prenatal sex steroids. However, one trait, the anogenital distance (AGD), may well be influenced by prenatal T, but shows little or no correlation with 2D:4D in rodents [for mice, see Ref. (13, 14); for rats, see Ref. (15)]. Why is this so? In humans the sex difference in 2D:4D is of medium effect size (5). It is determined toward the end of the first trimester of pregnancy (6, 7) in a narrow time window [for mice see Ref. (14)] and its magnitude changes little with subsequent growth (8, 9). In contrast, AGD shows a large sex difference, which varies from 1.4-fold-longer in males at 11–13 weeks to 2.0-fold-longer at 17–20 week gestation and a smaller difference is found in adults (16). This variability suggests that – unlike 2D:4D – the sex difference in AGD is not fixed early *in utero* but is influenced by fluctuations in second-trimester and post-natal androgens. Indeed, there has been one study in mice that experimentally confirmed these suggested effects [see Ref. (4, 14) for discussion]. There have been reports that both AGD and 2D:4D change when fetuses are exposed to endocrine disruptors [AGD, see Ref. (16); 2D:4D, see Ref. (15)]. The study by Auger et al. is of particular relevance here, as these authors compared the effect of estrogenic and anti-androgenic compounds on 2D:4D and AGD in rats. The authors reported a feminization effect for 2D:4D, but not so for AGD, which again suggests different times of developmental fixation of the sexual dimorphism in 2D:4D and AGD.

Correlational studies have also focused on the relationship between 2D:4D and the structure of the androgen receptor gene (AR), with emphasis on the number of CAG repeats in the AR.

Sensitivity to T is negatively associated to CAGn, such that in general population samples the highest sensitivity is found for CAGn of about 10 and lowest sensitivity for CAGn of about 30. Therefore, we might expect that 2D:4D is positively correlated with CAGn. There is mixed evidence from studies that have investigated this relationship and a recent meta-analysis including 14 samples and 1904 participants found no association between 2D:4D and CAGn (17). However, a closer inspection of the link between CAGn and T-dependent phenotypic traits suggests that normal variability of CAGn has mostly no, very small, or inconsistent effects [for example see Ref. AGD; (18)]. Thus, Hönekopp (17) concluded that “the lack of a clear correlation between CAGn and 2D:4D has no negative implications for the latter’s validity as a marker of prenatal testosterone effects.”

Experimental studies of the effects of FT and FE on 2D:4D are based on the assumption that the effects of prenatal hormones on human 2D:4D are essentially similar to those observed in other mammals. Consistent with this assumption, the 2D:4D of mammals, such as chimpanzees and bonobos (19), mice (20, 21), and rats (15) have been reported to show a sexual dimorphism, which is similar to that observed in humans (i.e., lower 2D:4D in males compared to females). In addition, comparative studies of primates showed that selection for high FT, resulting from a polygynous mating system, leads to the evolution of low 2D:4D (22). Moreover, the manipulation of FT and FE in animal models provides persuasive evidence for the developmental origins of 2D:4D. In rodents, there have been three such studies. Talarovicova et al. (23) reported that maternal enhancement of PT during pregnancy increased 4D length and reduced 2D:4D in both male and female rats. Zheng and Cohn (14) found that in mice a balance of FT to FE controlled 2D:4D, such that high FT increased 4D (leading to a reduction in 2D:4D) and high FE reduced 4D (leading to an increase in 2D:4D). The ratio of FT/FE had a marked effect on 4D because this fetal digit was richly supplied with receptors for FT and FE. Hence, Zheng and Cohn concluded that “digit ratio is a lifelong signal of prenatal hormonal exposure.” This model was developed further by Auger et al. (15) who exposed rat fetuses to environmental levels of estrogenic and anti-androgenic disruptors. They found that, in comparison to controls, such disruptors feminized digit ratios in male rats and concluded 2D:4D was a biomarker of prenatal exposure to low-dose environmental levels of endocrine disruptors.

## Right–Left Differences in 2D:4D

Zheng and Cohn’s (14) study has been influential in clarifying the relationship between 2D:4D and T/E ratios in the fetus. It has also shed some light on right–left differences (Dr–l) and associated effects on sex differences in 2D:4D. In their initial study, Manning et al. (2) reported that in humans right 2D:4D showed stronger relationships with target traits (such as T, E, and sperm numbers) than did left 2D:4D, suggesting that right 2D:4D is more sensitive to prenatal sex steroids than left 2D:4D [(2); see also Ref. (3), p.21]. More recently, Hönekopp and Watson (5) reported that the sex difference in right 2D:4D was greater than that of left 2D:4D. In order to determine whether 2D and 4D length is specified differently in males and females, Zheng and Cohn (14) used expression of Sox9, the earliest molecular marker of cartilage differentiation, to label the primordium of each digit in a sample of mice. They then measured the length of the Sox9 domain in the second and fourth rays. At embryonic day (ED) 12.5, when cartilage condensations first appeared, males and females did not differ significantly in 2D:4D, indicating that the sexual dimorphism arises after condensation of the digit primordia. By ED17, however, a significant sex difference in 2D:4D had emerged in the right hind paw. Of interest here, the left hind paw showed no significant difference between males and females, which is similar to the right–left asymmetry in adult humans; however, in mice both right and left hind paws exhibited a significant dimorphism by P2. These results indicate that the sexual dimorphism of 2D:4D in mice develops during a narrow window of embryonic development and that 2D:4D of the right paw is, at least initially, more sensitive to prenatal sex steroids than 2D:4D of the left paw [see also Ref. (4)]. Studies of 2D:4D usually consider both right and left 2D:4D, but also right–left 2D:4D or Dr–l as an additional negative marker for prenatal testosterone and a positive marker for prenatal estrogen (24, 25).

## 2D:4D and Adult Testosterone and Estrogen

Digit ratio has been reported to show associations with a number of behavioral and morphological traits expressed in adults (3, 26). This is likely to be because 2D:4D is supposed to be a biomarker for the organizing effects of FT and FE, although this conclusion does not explain how such organizing effects influence adult traits. It may be that 2D:4D is in some way associated with adult levels of T and E through its links with FT and FE. However, most studies suggest that 2D:4D correlations with adult T and E provide at best a very faint echo of associations between 2D:4D and FT/FE. A few studies have reported a negative correlation between 2D:4D and T [e.g., Ref. (2, 27, 28)] and a positive correlation with E [e.g., Ref. (2, 29)]. Yet a review of the evidence (30) and a meta-analysis (31) concluded that in the normal non-clinical population 2D:4D is not associated with adult sex hormone levels. This view was supported by a recent study of 1036 men and 620 women aged between 39 and 70 years (32). As predicted, T and T/E ratio was found to be significantly negatively related to right 2D:4D in men, but the effects were very weak. There were no significant associations in women. In conclusion, this large study confirmed that 2D:4D is negatively associated with high T in men but the association is weak and no more than an echo of the prenatal position.

## 2D:4D and “Challenge” Associated Spikes in Testosterone

Testosterone has energetic and immunosuppressive costs and maintaining high resting levels in the absence of challenges may be maladaptive (33, 34). Therefore, T levels tend to show spikes in response to “challenges,” such as aggressive (35) and sexual (36) encounters and to competitive sports such as soccer (37). It is now becoming clear that improved muscular performance results from such spikes (38, 39). Based on the literature on relationships of 2D:4D with aggression and sports performance, we suggest that 2D:4D is not related to resting T but is associated with the magnitude, and perhaps the response to, T spikes.

There have been two studies that have considered 2D:4D and T spikes induced by exercise or by an aggressive video. (i) The participants in the former were 79 professional rugby union players. Of these, 54 players served as controls and 25 were challenged using a repeated sprint agility test. In the experimental group T was measured immediately before the test and 5 and 20 min after completion. It was found that players with low right 2D:4D relative to left 2D:4D (low Dr–l) produced the highest amount of T at all three time points. The controls showed no association between 2D:4D and testosterone (24). (ii) With regard to the latter study, 45 participants were exposed to an aggressive video (rugby tackles and a “*haka*”) and a control video (a blank screen). Testosterone was assayed before and after each video and an aggression questionnaire completed after each video. The aggressive video was associated with a marginally significant increase in T, but the control video was not. Low 2D:4D (this time left 2D:4D) predicted high aggression scores after the aggressive video, and the association was particularly strong in participants showing the highest increase in T. However, there were no associations between 2D:4D and aggression after the control video (25).

If low 2D:4D predicts high T spikes in response to competition in aggressive sports and to aggressive stimuli then we may expect that low 2D:4D is associated with performance in many competitive sports.

## 2D:4D and the “Challenge” Link with Sports

Digit ratio shows relationships with many traits, but the effect sizes of reported associations are far from uniform. For example, in sports there are considerable relationships with negative correlations of about 0.4–0.6 reported in some sports such as distance running, rowing, rugby, and surfing, but also weak associations in sprinting and strength events [see Ref. (40) for a meta-analysis]. We suggest that many of these associations are driven by the link between low 2D:4D and pronounced spikes of T after challenge. For example, it is known that low Dr–l is predictive of high performance in elite rugby union players, with low Dr–l associated with high representation at international level (number of “caps”) and high number of tries scored (41). Low Dr–l is also a predictor of high T spikes, and this correlation may underlie the link between 2D:4D and sports. There are also behavioral traits, which are associated with 2D:4D, but in general the effect sizes of such relationships are much weaker than the link with sports. An appropriate and related example is aggression. Aggression is important in many sports, and low 2D:4D has been reported to be correlated with high physical aggression. However, in a non-sporting context the association is generally considered to be weak and requires large sample sizes to be demonstrated convincingly (5, 42). Given this dichotomy of results, we suggest that low 2D:4D is robustly linked to high aggression, but it is the context in which aggression is measured that is important here. The work of Millet (43) illustrates this inter-actionist perspective, suggesting that low 2D:4D does predict high aggression if the participants are subject to provocation or are placed in a threatening context (43, 44). In such situations, we expect marked spikes in T and robust correlations between 2D:4D and aggression. However, if participants are tested in neutral conditions then links between 2D:4D and aggression should be tenuous. Another example of such context-dependent findings is the intensely competitive environment of short-term financial trading. We should not be surprised that in this setting a strong negative correlation between 2D:4D and financial success was reported (45). On the contrary, in neutral laboratory conditions the links between 2D:4D and aggression are typically much weaker and should be seen with caution (46).

Studies on the associations between 2D:4D and sport are often focused on male participants. However, there is some evidence that low 2D:4D is linked to high levels of performance in females also (40), yet the overall picture with regard to 2D:4D in female athletes remains obscure [e.g., for handgrip strength, see Ref. (47)]. Little is known with regard to “challenge-related” spikes of T in females, although there is some support for a link between 2D:4D and sensitivity to administered T in women. In three studies, Van Honk and colleagues have shown that administered T modulates empathy (48), cooperation (49), and moral judgments (50) in women, and that 2D:4D strongly moderates the effects. The sample sizes in these studies are small, but the effect sizes are very large with 2D:4D explaining 25–44% of the variance of the effects of T. It is yet unclear why 2D:4D predicts response to administered spikes in T in such studies, but similar relationships between 2D:4D and response to T may also be found in men.

## Conclusion

We conclude that 2D:4D is a biomarker for the balance between FT and FE, such that high FT and low FE is linked to low 2D:4D. There is evidence that 2D:4D is fixed in a relatively narrow developmental window at the end of the first trimester of pregnancy and that it does not change substantially with age. Considering 15 years of work on this topic, we feel that there is quite strong evidence for this link. However, more contentiously, we hypothesize that the relative levels of FT and FE have organizing effects on the adult endocrine system, which are particularly evident in “challenging” situations, such as aggressive or sexual encounters. This means that 2D:4D should correlate with T spikes produced under challenge and it may also be linked to response to such spikes. In consequence, low 2D:4D may be a predictor of high performance in sports and high aggression when provoked. We suggest that future studies regarding the links between 2D:4D and such traits as adult T levels, sports performance, and aggression should include aggressive and/or sexual stimuli in their protocols. In this way our hypothesized link between 2D:4D and T spikes may be tested.

## Conflict of Interest Statement

The authors declare that the research was conducted in the absence of any commercial or financial relationships that could be construed as a potential conflict of interest.
